# *N*-Benzoyl-2-nitro­benzene­sulfonamide

**DOI:** 10.1107/S1600536811055917

**Published:** 2012-01-11

**Authors:** P. A. Suchetan, Sabine Foro, B. Thimme Gowda, B. Nirmala

**Affiliations:** a Department of Chemistry, Mangalore University, Mangalagangotri 574 199, Mangalore, India; b Institute of Materials Science, Darmstadt University of Technology, Petersenstrasse 23, D-64287 Darmstadt, Germany; c Department of Chemistry, University College of Science, Tumkur University, Tumkur 572 102, India

## Abstract

In the title compound, C_13_H_10_N_2_O_5_S, the N—C bond in the C—SO_2_—NH—C segment has *gauche* torsion angles with respect to the S=O bonds. The conformation between the N—H bond and the *ortho*-nitro group in the sulfonyl benzene ring is *syn*. The mol­ecule is twisted at the S—N bond with a torsion angle of −63.4 (2)°. The sulfonyl benzene ring is tilted by 77.1 (1)° relative to the —SO_2_—NH—C—O segment. The dihedral angle between the sulfonyl and the benzoyl benzene rings is 88.6 (1)°. In the crystal, pairs of N—H⋯O(S) hydrogen bonds link the mol­ecules into inversion dimers, which are linked by weak C—H⋯O and C—H⋯π inter­actions along the *b* axis.

## Related literature

For studies, including those by our group, on the effects of substituents on the structures and other aspects of *N*-(ar­yl)-amides, see: Bowes *et al.* (2003 ▶); Gowda *et al.* (2006 ▶), on *N*-(ar­yl)-methane­sulfonamides, see: Jayalakshmi & Gowda (2004 ▶), on *N*-(ar­yl)-aryl­sulfonamides, see: Gowda *et al.* (2003 ▶), on *N*-(substitutedbenzo­yl)-aryl­sulfonamides, see: Suchetan *et al.* (2010 ▶) and on *N*-chloro­aryl­amides, see: Gowda & Maha­de­vappa (1983 ▶).
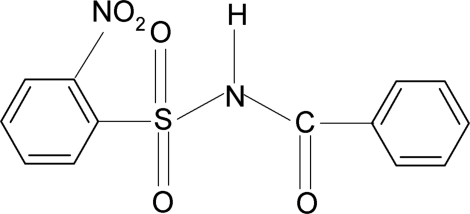


## Experimental

### 

#### Crystal data

C_13_H_10_N_2_O_5_S
*M* *_r_* = 306.29
Orthorhombic, 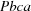

*a* = 12.1127 (8) Å
*b* = 11.7625 (8) Å
*c* = 18.730 (1) Å
*V* = 2668.6 (3) Å^3^
*Z* = 8
Mo *K*α radiation
μ = 0.27 mm^−1^
*T* = 293 K
0.48 × 0.44 × 0.40 mm

#### Data collection

Oxford Diffraction Xcalibur diffractometer with a Sapphire CCD detector
Absorption correction: multi-scan (*CrysAlis RED*; Oxford Diffraction, 2009 ▶) *T*_min_ = 0.883, *T*_max_ = 0.901
6396 measured reflections
2711 independent reflections
2010 reflections with *I* > 2σ(*I*)
*R*_int_ = 0.020

#### Refinement

*R*[*F* ^2^ > 2σ(*F* ^2^)] = 0.043
*wR*(*F* ^2^) = 0.110
*S* = 1.04
2711 reflections
193 parameters
1 restraint
H atoms treated by a mixture of independent and constrained refinement
Δρ_max_ = 0.31 e Å^−3^
Δρ_min_ = −0.36 e Å^−3^

### 

Data collection: *CrysAlis CCD* (Oxford Diffraction, 2009 ▶); cell refinement: *CrysAlis RED* (Oxford Diffraction, 2009 ▶); data reduction: *CrysAlis RED*; program(s) used to solve structure: *SHELXS97* (Sheldrick, 2008 ▶); program(s) used to refine structure: *SHELXL97* (Sheldrick, 2008 ▶); molecular graphics: *PLATON* (Spek, 2009 ▶); software used to prepare material for publication: *SHELXL97*.

## Supplementary Material

Crystal structure: contains datablock(s) I, global. DOI: 10.1107/S1600536811055917/bq2330sup1.cif

Structure factors: contains datablock(s) I. DOI: 10.1107/S1600536811055917/bq2330Isup2.hkl

Supplementary material file. DOI: 10.1107/S1600536811055917/bq2330Isup3.cml

Additional supplementary materials:  crystallographic information; 3D view; checkCIF report

## Figures and Tables

**Table 1:** Hydrogen-bond geometry (Å, °) *Cg*1 is the centroid of the C8–C13 ring.

*D*—H⋯*A*	*D*—H	H⋯*A*	*D*⋯*A*	*D*—H⋯*A*
N1—H1*N*⋯O2^i^	0.84 (2)	2.21 (2)	3.003 (3)	158 (2)
C11—H11⋯O3^ii^	0.93	2.51	3.267 (3)	139
C13—H13⋯O2^i^	0.93	2.53	3.313 (3)	142
C6—H6⋯*Cg*1^iii^	0.93	2.82	3.678 (13)	153

Symmetry codes: (i) 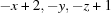; (ii) 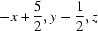; (iii) 
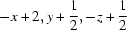
.

**Fig. 1.**
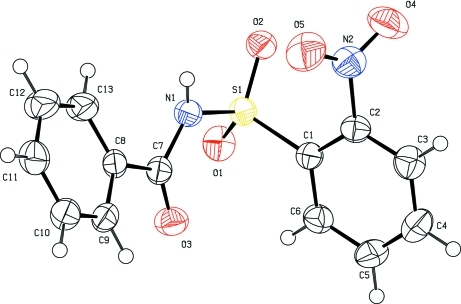
Molecular structure of the title compound, showing the atom-labeling scheme. Displacement ellipsoids are drawn at the 50% probability level.

**Fig. 2.**
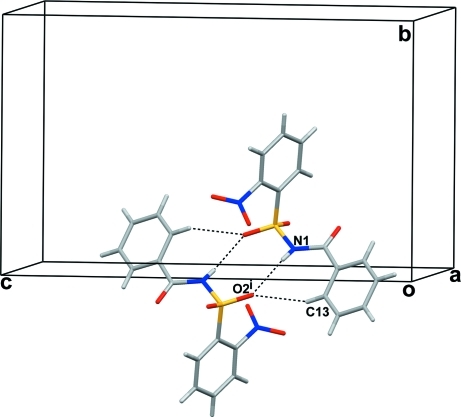
Part of the crystal structure of the title compound, showing the dimers formed by N-H···O and C-H..O hydrogen bonds. [Symmetry code: (i) 2-x, -y, 1-z]

**Fig. 3.**
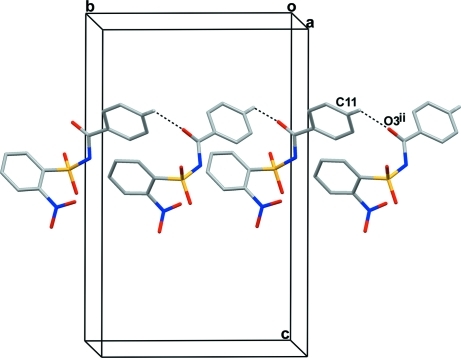
Part of the crystal structure of the title compound, showing the linking of the dimers by C-H···O hydrogen bonds along to the [010] direction. For the sake of clarity, H atoms not involved in the motif shown have been omitted. [Symmetry code: (ii) = 5/2-x, y-1/2, z]

**Fig. 4.**
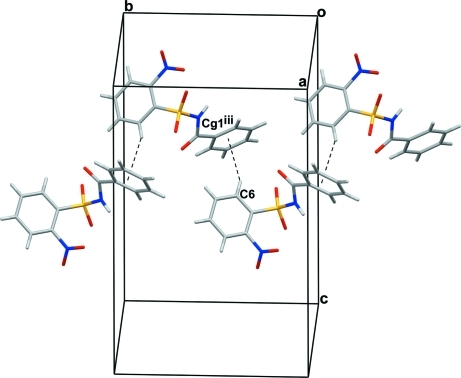
Part of the crystal structure of the title compound, showing the formation of a chain along [010] generated by the C-H···π interactions. [Symmetry code: (iii) = 2-x, y+1/2, -z+1/2]

## supplementary crystallographic information

### Comment

Diaryl acylsulfonamides are known as potent antitumor agents against a broad spectrum of human tumor xenografts in nude mice. As part of our studies on the substituent effects on the structures and other aspects of *N*-(aryl)-amides (Bowes *et al.*, 2003; Gowda *et al.*, 2006), *N*-(aryl)-methanesulfonamides (Jayalakshmi & Gowda, 2004), *N*-(aryl)-arylsulfonamides (Gowda *et al.*, 2003); *N*-(substitutedbenzoyl)-arylsulfonamides (Suchetan *et al.*, 2010) and *N*-chloro-arylsulfonamides (Gowda & Mahadevappa, 1983), in the present work, the crystal structure of *N*-(benzoyl)- 2-nitrobenzenesulfonamide (I) has been determined (Fig.1).

In (I), the conformation between the N—H and C=O bonds in the C—SO_2_—NH—C(O) segment is *anti* (Fig.1), similar to that observed in *N*-(benzoyl)-2-methylbenzenesulfonamide (II) (Suchetan *et al.*, 2010). Furthermore, the N—C bond in the segment has *gauche* torsion with respect to the S═O bonds, while, the conformation between the N—H bond and the *ortho*-nitro group in the sulfonyl benzene ring is *syn*.

The molecule is twisted at the S—N bond with the torsional angle of -63.39 (22)°, compared to the value of 68.8 (4)° in (II).

The dihedral angle between the sulfonyl benzene ring and the —SO_2_—NH—C—O segment is 77.1 (1)°, compared to the value of 84.8 (1)° in (II). Further, the dihedral angle between the sulfonyl and the benzoyl benzene rings is 88.6 (1)°, compared to the value of 73.9 (1)° in (II).

In the crystal, intermolecular N1–H1N···O2 and C13—H13···O2 hydrogen bonds link the molecules as dimers and these dimers are also linked by C11—H11···O3 hydrogen bonds and C—H···π interactions along b-axis (Table 1). [C6—H6···*Cg*1, cg1 is the centroid of C8—C13 ring] Parts of the crystal structure are shown in Fig. 2., Fig. 3. and Fig. 4.

### Experimental

The title compound was prepared by refluxing a mixture of benzoic acid (0.02 mole), 2-nitrobenzenesulfonamide (0.02 mole) and excess phosphorous oxy chloride for 3 h on a water bath. The resultant mixture was cooled and poured into crushed ice. The solid, *N*-(benzoyl)-2-nitrobenzenesulfonamide, obtained was filtered, washed thoroughly with water and then dissolved in sodium bicarbonate solution. The compound was later reprecipitated by acidifying the filtered solution with dilute HCl. It was filtered, dried and recrystallized.

Prism like colourless single crystals of the title compound used in X-ray diffraction studies were obtained by slow evaporation of its toluene solution at room temperature.

### Refinement

The H atom of the NH group was located in a difference map and later restrained to N—H = 0.86 (2) Å. The other H atoms were positioned with idealized geometry using a riding model with C—H = 0.93 Å. All H atoms were refined with isotropic displacement parameters (set to 1.2 times of the *U*_eq_ of the parent atom).

### Crystal data

**Table:**

C_13_H_10_N_2_O_5_S	*F*(000) = 1264
*M**_r_* = 306.29	*D*_x_ = 1.525 Mg m^−^^3^
Orthorhombic, *P**b**c**a*	Mo *K*α radiation, λ = 0.71073 Å
Hall symbol: -P 2ac 2ab	Cell parameters from 2597 reflections
*a* = 12.1127 (8) Å	θ = 2.6–27.8°
*b* = 11.7625 (8) Å	µ = 0.27 mm^−^^1^
*c* = 18.730 (1) Å	*T* = 293 K
*V* = 2668.6 (3) Å^3^	Prism, colorless
*Z* = 8	0.48 × 0.44 × 0.40 mm

### Data collection

**Table:**

Oxford Diffraction Xcalibur diffractometer with a Sapphire CCD detector	2711 independent reflections
Radiation source: fine-focus sealed tube	2010 reflections with *I* > 2σ(*I*)
graphite	*R*_int_ = 0.020
Rotation method data acquisition using ω scans	θ_max_ = 26.4°, θ_min_ = 2.7°
Absorption correction: multi-scan (*CrysAlis RED*; Oxford Diffraction, 2009)	*h* = −13→15
*T*_min_ = 0.883, *T*_max_ = 0.901	*k* = −14→14
6396 measured reflections	*l* = −14→23

### Refinement

**Table:**

Refinement on *F*^2^	Primary atom site location: structure-invariant direct methods
Least-squares matrix: full	Secondary atom site location: difference Fourier map
*R*[*F*^2^ > 2σ(*F*^2^)] = 0.043	Hydrogen site location: inferred from neighbouring sites
*wR*(*F*^2^) = 0.110	H atoms treated by a mixture of independent and constrained refinement
*S* = 1.04	*w* = 1/[σ^2^(*F*_o_^2^) + (0.0388*P*)^2^ + 2.8009*P*] where *P* = (*F*_o_^2^ + 2*F*_c_^2^)/3
2711 reflections	(Δ/σ)_max_ = 0.001
193 parameters	Δρ_max_ = 0.31 e Å^−^^3^
1 restraint	Δρ_min_ = −0.36 e Å^−^^3^

### Special details

**Table:**

Experimental. CrysAlis RED (Oxford Diffraction, 2009) Empirical absorption correction using spherical harmonics, implemented in SCALE3 ABSPACK scaling algorithm.
Geometry. All e.s.d.'s (except the e.s.d. in the dihedral angle between two l.s. planes) are estimated using the full covariance matrix. The cell e.s.d.'s are taken into account individually in the estimation of e.s.d.'s in distances, angles and torsion angles; correlations between e.s.d.'s in cell parameters are only used when they are defined by crystal symmetry. An approximate (isotropic) treatment of cell e.s.d.'s is used for estimating e.s.d.'s involving l.s. planes.
Refinement. Refinement of *F*^2^ against ALL reflections. The weighted *R*-factor *wR* and goodness of fit *S* are based on *F*^2^, conventional *R*-factors *R* are based on *F*, with *F* set to zero for negative *F*^2^. The threshold expression of *F*^2^ > σ(*F*^2^) is used only for calculating *R*-factors(gt) *etc*. and is not relevant to the choice of reflections for refinement. *R*-factors based on *F*^2^ are statistically about twice as large as those based on *F*, and *R*- factors based on ALL data will be even larger.

### Fractional atomic coordinates and isotropic or equivalent isotropic displacement parameters (Å^2^)

**Table:**

	*x*	*y*	*z*	*U*_iso_*/*U*_eq_	
C1	1.01092 (19)	0.2867 (2)	0.42850 (12)	0.0320 (5)	
C2	1.08964 (19)	0.3142 (2)	0.47986 (13)	0.0352 (5)	
C3	1.1400 (2)	0.4186 (2)	0.48146 (15)	0.0458 (6)	
H3	1.1912	0.4360	0.5168	0.055*	
C4	1.1134 (2)	0.4975 (2)	0.42959 (15)	0.0503 (7)	
H4	1.1473	0.5684	0.4298	0.060*	
C5	1.0369 (2)	0.4717 (2)	0.37750 (15)	0.0496 (7)	
H5	1.0201	0.5249	0.3424	0.060*	
C6	0.9851 (2)	0.3670 (2)	0.37733 (14)	0.0413 (6)	
H6	0.9326	0.3505	0.3426	0.050*	
C7	1.0522 (2)	0.0708 (2)	0.31993 (13)	0.0344 (5)	
C8	1.13311 (19)	−0.01632 (19)	0.29607 (12)	0.0332 (5)	
C9	1.2011 (2)	0.0112 (2)	0.23910 (13)	0.0400 (6)	
H9	1.1942	0.0816	0.2170	0.048*	
C10	1.2786 (2)	−0.0652 (2)	0.21516 (14)	0.0436 (6)	
H10	1.3247	−0.0458	0.1773	0.052*	
C11	1.2882 (2)	−0.1697 (2)	0.24688 (14)	0.0482 (7)	
H11	1.3412	−0.2209	0.2309	0.058*	
C12	1.2194 (3)	−0.1986 (2)	0.30227 (16)	0.0589 (8)	
H12	1.2252	−0.2701	0.3231	0.071*	
C13	1.1419 (2)	−0.1227 (2)	0.32734 (14)	0.0482 (7)	
H13	1.0959	−0.1428	0.3650	0.058*	
N1	1.01881 (17)	0.06273 (17)	0.39120 (11)	0.0355 (5)	
H1N	1.051 (2)	0.021 (2)	0.4206 (12)	0.043*	
N2	1.12183 (18)	0.2319 (2)	0.53570 (13)	0.0467 (6)	
O1	0.84056 (14)	0.17061 (15)	0.38774 (10)	0.0455 (5)	
O2	0.92831 (15)	0.11935 (15)	0.50171 (9)	0.0418 (4)	
O3	1.01759 (16)	0.14595 (15)	0.28213 (9)	0.0468 (5)	
O4	1.09678 (19)	0.2545 (2)	0.59685 (11)	0.0660 (6)	
O5	1.1726 (2)	0.14835 (19)	0.51681 (13)	0.0695 (6)	
S1	0.93749 (5)	0.15669 (5)	0.42926 (3)	0.03293 (17)	

### Atomic displacement parameters (Å^2^)

**Table:**

	*U*^11^	*U*^22^	*U*^33^	*U*^12^	*U*^13^	*U*^23^
C1	0.0320 (11)	0.0317 (12)	0.0323 (12)	0.0037 (10)	0.0015 (10)	0.0001 (10)
C2	0.0327 (12)	0.0375 (13)	0.0355 (12)	0.0042 (10)	−0.0013 (10)	0.0035 (10)
C3	0.0393 (14)	0.0489 (16)	0.0492 (15)	−0.0016 (13)	−0.0060 (12)	−0.0019 (13)
C4	0.0541 (16)	0.0358 (14)	0.0612 (17)	−0.0060 (13)	0.0042 (15)	0.0024 (13)
C5	0.0635 (18)	0.0386 (15)	0.0468 (15)	0.0050 (14)	−0.0012 (14)	0.0098 (12)
C6	0.0469 (14)	0.0410 (14)	0.0360 (13)	0.0046 (12)	−0.0051 (12)	0.0010 (11)
C7	0.0372 (13)	0.0313 (12)	0.0347 (12)	−0.0031 (11)	0.0015 (11)	−0.0029 (10)
C8	0.0365 (12)	0.0312 (12)	0.0317 (12)	−0.0033 (11)	0.0017 (10)	−0.0058 (10)
C9	0.0483 (14)	0.0309 (12)	0.0408 (14)	−0.0057 (11)	0.0069 (12)	−0.0018 (11)
C10	0.0445 (15)	0.0418 (14)	0.0444 (14)	−0.0036 (12)	0.0145 (12)	−0.0040 (12)
C11	0.0536 (16)	0.0444 (15)	0.0465 (15)	0.0130 (13)	0.0086 (13)	−0.0081 (13)
C12	0.087 (2)	0.0385 (15)	0.0512 (16)	0.0179 (16)	0.0180 (16)	0.0066 (13)
C13	0.0654 (18)	0.0397 (14)	0.0395 (14)	0.0059 (14)	0.0193 (13)	0.0026 (12)
N1	0.0412 (12)	0.0330 (11)	0.0324 (11)	0.0076 (9)	0.0019 (9)	0.0004 (9)
N2	0.0417 (12)	0.0494 (14)	0.0490 (14)	0.0001 (11)	−0.0116 (11)	0.0062 (11)
O1	0.0335 (9)	0.0494 (11)	0.0535 (11)	0.0024 (8)	−0.0053 (8)	−0.0030 (9)
O2	0.0489 (10)	0.0402 (10)	0.0361 (9)	0.0012 (8)	0.0105 (8)	0.0004 (8)
O3	0.0561 (11)	0.0424 (10)	0.0420 (10)	0.0097 (9)	0.0068 (9)	0.0063 (9)
O4	0.0764 (15)	0.0815 (16)	0.0400 (12)	−0.0017 (13)	−0.0101 (11)	0.0097 (11)
O5	0.0756 (15)	0.0536 (13)	0.0794 (15)	0.0219 (12)	−0.0179 (13)	0.0069 (12)
S1	0.0321 (3)	0.0332 (3)	0.0335 (3)	0.0027 (3)	0.0028 (3)	−0.0015 (2)

### Geometric parameters (Å, °)

**Table:**

C1—C6	1.381 (3)	C8—C13	1.386 (3)
C1—C2	1.392 (3)	C9—C10	1.375 (3)
C1—S1	1.769 (2)	C9—H9	0.9300
C2—C3	1.372 (4)	C10—C11	1.370 (4)
C2—N2	1.477 (3)	C10—H10	0.9300
C3—C4	1.382 (4)	C11—C12	1.374 (4)
C3—H3	0.9300	C11—H11	0.9300
C4—C5	1.379 (4)	C12—C13	1.377 (4)
C4—H4	0.9300	C12—H12	0.9300
C5—C6	1.382 (4)	C13—H13	0.9300
C5—H5	0.9300	N1—S1	1.643 (2)
C6—H6	0.9300	N1—H1N	0.835 (17)
C7—O3	1.207 (3)	N2—O5	1.211 (3)
C7—N1	1.398 (3)	N2—O4	1.214 (3)
C7—C8	1.487 (3)	O1—S1	1.4177 (18)
C8—C9	1.386 (3)	O2—S1	1.4308 (17)
			
C6—C1—C2	118.4 (2)	C8—C9—H9	119.8
C6—C1—S1	118.86 (19)	C11—C10—C9	120.2 (2)
C2—C1—S1	122.61 (18)	C11—C10—H10	119.9
C3—C2—C1	121.8 (2)	C9—C10—H10	119.9
C3—C2—N2	117.0 (2)	C10—C11—C12	119.9 (2)
C1—C2—N2	121.2 (2)	C10—C11—H11	120.1
C2—C3—C4	118.8 (3)	C12—C11—H11	120.1
C2—C3—H3	120.6	C11—C12—C13	120.7 (3)
C4—C3—H3	120.6	C11—C12—H12	119.7
C5—C4—C3	120.4 (3)	C13—C12—H12	119.7
C5—C4—H4	119.8	C12—C13—C8	119.6 (2)
C3—C4—H4	119.8	C12—C13—H13	120.2
C4—C5—C6	120.2 (3)	C8—C13—H13	120.2
C4—C5—H5	119.9	C7—N1—S1	122.79 (17)
C6—C5—H5	119.9	C7—N1—H1N	122.4 (18)
C1—C6—C5	120.3 (2)	S1—N1—H1N	112.9 (18)
C1—C6—H6	119.8	O5—N2—O4	125.5 (2)
C5—C6—H6	119.8	O5—N2—C2	117.3 (2)
O3—C7—N1	120.7 (2)	O4—N2—C2	117.3 (2)
O3—C7—C8	123.8 (2)	O1—S1—O2	119.43 (11)
N1—C7—C8	115.5 (2)	O1—S1—N1	109.64 (11)
C9—C8—C13	119.4 (2)	O2—S1—N1	104.57 (10)
C9—C8—C7	117.5 (2)	O1—S1—C1	108.18 (11)
C13—C8—C7	123.1 (2)	O2—S1—C1	108.18 (11)
C10—C9—C8	120.3 (2)	N1—S1—C1	106.06 (11)
C10—C9—H9	119.8		
			
C6—C1—C2—C3	1.2 (4)	C10—C11—C12—C13	−1.2 (5)
S1—C1—C2—C3	−175.2 (2)	C11—C12—C13—C8	0.3 (5)
C6—C1—C2—N2	−179.4 (2)	C9—C8—C13—C12	1.2 (4)
S1—C1—C2—N2	4.2 (3)	C7—C8—C13—C12	−179.8 (3)
C1—C2—C3—C4	−1.5 (4)	O3—C7—N1—S1	−3.5 (3)
N2—C2—C3—C4	179.1 (2)	C8—C7—N1—S1	175.16 (16)
C2—C3—C4—C5	0.4 (4)	C3—C2—N2—O5	−112.1 (3)
C3—C4—C5—C6	0.8 (4)	C1—C2—N2—O5	68.4 (3)
C2—C1—C6—C5	0.1 (4)	C3—C2—N2—O4	66.7 (3)
S1—C1—C6—C5	176.7 (2)	C1—C2—N2—O4	−112.7 (3)
C4—C5—C6—C1	−1.1 (4)	C7—N1—S1—O1	53.2 (2)
O3—C7—C8—C9	23.9 (4)	C7—N1—S1—O2	−177.62 (19)
N1—C7—C8—C9	−154.7 (2)	C7—N1—S1—C1	−63.4 (2)
O3—C7—C8—C13	−155.1 (3)	C6—C1—S1—O1	−16.5 (2)
N1—C7—C8—C13	26.2 (3)	C2—C1—S1—O1	159.83 (19)
C13—C8—C9—C10	−1.8 (4)	C6—C1—S1—O2	−147.3 (2)
C7—C8—C9—C10	179.1 (2)	C2—C1—S1—O2	29.1 (2)
C8—C9—C10—C11	0.9 (4)	C6—C1—S1—N1	101.0 (2)
C9—C10—C11—C12	0.7 (4)	C2—C1—S1—N1	−82.6 (2)

### Hydrogen-bond geometry (Å, °)

**Table:**

Cg1 is the centroid of the C8–C13 ring.

**Table:**

*D*—H···*A*	*D*—H	H···*A*	*D*···*A*	*D*—H···*A*
N1—H1N···O2^i^	0.84 (2)	2.21 (2)	3.003 (3)	158 (2)
C11—H11···O3^ii^	0.93	2.51	3.267 (3)	139
C13—H13···O2^i^	0.93	2.53	3.313 (3)	142
C6—H6···Cg1^iii^	0.93	2.82	3.678 (13)	153

Symmetry codes: (i) −*x*+2, −*y*, −*z*+1; (ii) −*x*+5/2, *y*−1/2, *z*; (iii) −*x*+2, *y*+1/2, −*z*+1/2.
